# Effect of Different Plants on the Growth and Reproduction of *Thrips flavus* (Thysanoptera: Thripidae)

**DOI:** 10.3390/insects12060502

**Published:** 2021-05-28

**Authors:** Yu Gao, Yijin Zhao, Di Wang, Jing Yang, Ning Ding, Shusen Shi

**Affiliations:** 1 College of Plant Protection, Jilin Agricultural University, Changchun 130118, China; dlzyjmm@163.com (Y.Z.); w1950215017@163.com (D.W.); dn960464198@163.com (N.D.); 2 College of Bio-Resource Science, Dankook University, Cheonan 31116, Korea; nevermore_yj@dankook.ac.kr

**Keywords:** *Thrips flavus*, population growth, development, host plant, life table

## Abstract

**Simple Summary:**

*Thrips flavus* Schrank (Thysanoptera: Thripidae) is a worldwide phytophagous pest in Palearctic Asian and European countries. *T. flavus* feeds on a wide spectrum of host plants. Thus, understanding its host plant preferences is important for pest control. We tested the development duration, population parameters, and population growth of *T. flavus* on five species of plants. The intrinsic rate of increase and fecundity was the highest on *Cucumis sativus*, followed by *Glycine max* and *Capsicum annuum*. However, *Solanum melongena* and *Brassica rapa* var. *glabra* were not suitable host plants. These results help to improve our understanding of the population dynamics of *T. flavus* and should lead to positive measures to control thrips in the field.

**Abstract:**

Host plants play an important role in affecting insect development and reproduction. Understanding the host plant preferences is important for pest control. *Thrips flavus* Schrank (Thysanoptera: Thripidae) is a worldwide phytophagous pest in Palearctic Asian and European countries. We used a life table analysis to study the development duration, population parameters, and population growth of *T. flavus* on five plant species, including *Solanum melongena* (Solanaceae), *Capsicum annuum* (Solanaceae), *Glycine max* (Leguminosae), *Brassica rapa* var. *glabra* (Cruciferae), and *Cucumis sativus* (Cucurbitaceae). The results showed that *T. flavus* can survive and reproduce on *Cu. sativus* and *G. max*, which were two potentially suitable host plants. *T. flavus* preferred to oviposit on *Cu. sativus* with a shorter duration of development (17.8 days) at 25 °C. Therefore, the host plant was an important factor influencing the development and fecundity of *T. flavus* populations. These results will improve our understanding of the population dynamics of *T. flavus* and facilitate the development of more scientific and efficient measures to control thrips.

## 1. Introduction

*Thrips flavus* Schrank (Thysanoptera: Thripidae) is a worldwide phytophagous insect in Palearctic Asian and European countries [1,2,3,4,5]. *T. flavus* feeds on a wide spectrum of host plants, including at least 97 species in 33 families, resulting in a reduction in the economic values of vegetables, fruits, flowers, tobacco, and oil crops [6,7]. The insects feed and oviposit on flowers, leaves, and pods causing direct damage [8,9,10]. The damage caused by *T. flavus* appears as necrotic silvering on the leaves and results in the curling, deformation and withering of the leaves and early senescence or deformation of the flowers [6]. Moreover, it has been reported that *T. flavus* transmits the tomato spotted wilt virus that infects watermelon (TSWV-W) [1,11]. *T. flavus* has well-developed mechanisms for its successful dispersion and may have the potential to cause large infestations [12]. *T. flavus* has been increasingly recognized as one of the main pests on soybean from the flowering stage to early stage of podding in Northeast China [13]. The results of studies in the field showed that the spatial distribution patterns of adults and nymphs were aggregated in each growth stage of soybean [14]. *T. flavus* is primarily distributed on the first to the fifth trifoliolate position of soybean plants and on the dorsal sides of leaves [15]. The management of *T. flavus* is heavily dependent on chemical control in this region [16]. It is generally believed that thrips are very difficult to control because of their developmental rate and resistance to insecticides [17,18]. It is critical to understand the biology of these pests on host plants to develop effective management strategies. Concurrently, it is known that the development and reproduction of thrips are significantly affected by host plants and many other environmental factors, such as temperature, humidity, and natural enemies [19,20]. However, the relationships between *T. flavus* and its host plants have not been thoroughly studied during the previous decade [1,6,13]. This study was conducted to determine the development and reproduction of *T. flavus* on five host plants (eggplant, pepper, soybean, Chinese cabbage, and cucumber) using a life table to provide basic data for a pest control strategy.

## 2. Materials and Methods

### 2.1. Plants and Insects

Five plants species that are common crops in Jilin Province, China, are eggplant, *Solanum melongena* (Solanaceae, cultivated variety: “Ziyu long-eggplant”); pepper, *Capsicum annuum* (Solanaceae, cultivated variety: “South Korean Jinfeng No.2”); soybean, *Glycine max* (Leguminosae, cultivated variety: “Jinong 38”); Chinese cabbage, *Brassica rapa* var. *glabra* (Cruciferae, cultivated variety: “Arctic spring No.3”); and cucumber, *Cucumis sativus* (Cucurbitaceae, cultivated variety: “Guamanjia”). The plants were planted in plastic pots (16.5 cm in diameter, 12 cm in the bottom diameter, and 12 cm high) in a separate room in a greenhouse (25 ± 2 °C and natural lighting) to prevent infection by pests and pathogens [21]. Seedling substrate (Tianyun Fertilizer Co., Ltd., Changchun, China) was applied, and the plants were then watered 2–5 times a week.

*T. flavus* individuals were collected from a cultivated soybean field in Jilin Province (43°48′10″ N, 125°24′35″ E) and were used to establish a laboratory colony. The tests were conducted from 15 May to 24 August 2020. Thrips were continuously reared on soybean trifoliolate leaves in transparent plastic containers (Φ 5 cm × 8 cm, 12 cm high) sealed by plastic film with micropores. The development and reproduction of *T. flavus* were observed at 25 ± 1 °C in an illuminated incubator (GXZ380B, Jiangnan Instrument Factory, Ningbo, China) with 70% ± 5% RH and a 16 h:8 h (L:D) photoperiod.

### 2.2. Development and Survival of Each Stage

A group of 100–150 female adults were placed in each plastic container that contained eggplant, pepper, soybean, Chinese cabbage, or cucumber for 24 h to allow oviposition. The leaves with eggs were moved into plastic petri dishes (10 cm diameter and 12 cm high). Wet cotton was used to keep the leaves alive. The newly hatched first instar nymph were observed daily under a stereomicroscope (SZ61, Olympus Corporation, Tokyo, Japan) at 8:00 and 20:00 until they became adults. The instar, developmental stage, sex of adults, and number of surviving individuals were recorded at each observation.

Each developmental stage was identified by the observation of exuviae in the culture dish [22] and defined as follows: (a) The egg stage was defined as the period from the beginning of egg laying to the time that the first instar nymphs were observed. (b) The first instar nymphs developed after exuviation, and the second instar nymphs developed after exuviating again. (c) The third instar nymphs were identified from two short wing-germs and two forwardly directed antennae. (d) The pupae (the forth instar nymph) have two long wing-germs and two backwardly directed antennae. (e) The adults have yellow bodies and formed wings. (f) The sex of adults was distinguished by an ovipositor in the female abdomen. The males were smaller than females, and their bodies were lighter in color [6].

### 2.3. Life Table Study

Life tables’ data were analyzed by a TWOSEX-MSChart program using the methods of Chi (2021) [23]. The population parameters were calculated based on Chi and Liu (1985) and Chi and Getz (1988) [24,25] using Equations (1) and (2):

(1) $$l_{x}=\sum_{j=1}^{k}s_{xj}$$

where *k* is the number of life stages, and

(2) $$m_{x}=\frac{\sum_{j=1}^{k}s_{xj}f_{xj}}{\sum_{j=1}^{k}s_{xj}}$$

The net reproductive rate (*R*_0_) is the total number of offspring that a female can produce during its lifetime. *R*_0_ was calculated using Equation (3) [21,26]:

(3) $$R_{0}=\sum_{x=0}^{\infty }l_{x}m_{x}$$

The intrinsic rate of increase (*r*) was used to express the ability of population to increase and was calculated using Equation (4) as the iterative bisection method from the Euler–Lotka equation [26]:

(4) $$\sum_{x=0}^{\infty }e^{-r_{m}(x+1)}l_{x}m_{x}=1$$

The finite rate of increase (*λ*) is a multiplication factor of the original population at each time period and was calculated using Equation (5) [21,26]:

(5) $$\lambda =e^{r}$$

The mean generation time (*T*) is defined as the length of time that a population requires to increase to *R*_0_—the approximate fold of its size when the population reaches a stable age stage. Its distribution was calculated using Equation (6) [21,26]:

(6) $$T=\frac{\ln(R_{0})}{r_{}}$$

The age-stage life expectancy (*e_xj_*) was determined using Equation (7) [27]:

(7) $$e_{xj}=\sum_{i=x}^{n}\sum_{j=y}^{m}{s^{\prime }}_{ij}$$

The age-stage reproductive value (*v_xj_*) was calculated using Equation (8) [26]:

(8) $$v_{xj}=\frac{e^{{}_{r(x+1)}}}{s_{xj}}\sum_{i=x}^{\infty }e^{-r(i+1)}\sum_{y=j}^{k}{s^{\prime }}_{iy}f_{iy}$$

The population trend index (*I*) was determined using Equation (9) based on the life table data [27]:

(9) $$I=\frac{N_{1}}{N_{0}}$$

where *N*_0_ is the amount of eggs of the initial generation, and *N*_1_ is the amount of eggs of the following generation.

### 2.4. Statistical Analysis

The data were analyzed using PASW Statistics V18.0 (IBM, New York, NY, USA). A one-way analysis of variance (ANOVA) followed by Duncan’s new multiple range test was used to ascertain the significance of differences in developmental durations on different plants.

## 3. Results

### 3.1. Developmental Durations and Survival Rates

**Figure 1:**
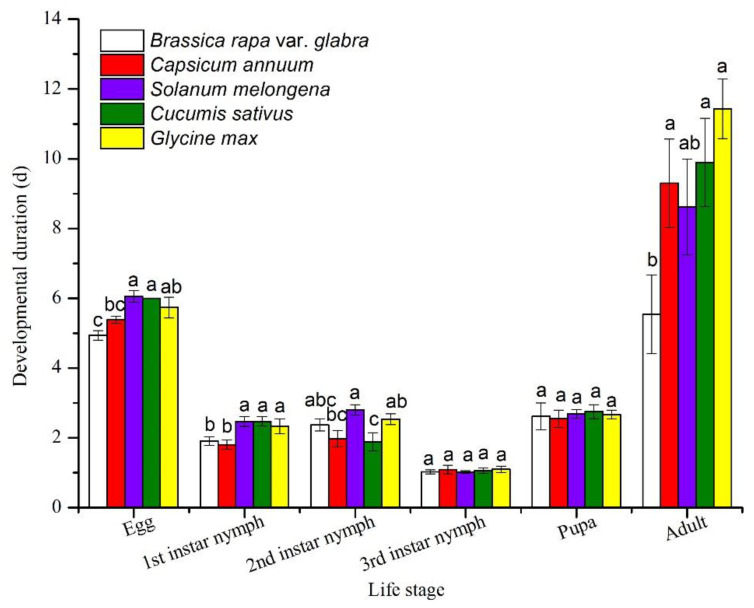
Developmental durations of *Thrips flavus* fed on different plants. Different letters indicate significant differences among different treatments at the 0.05 level.

The developmental duration of eggs was the highest on eggplant (6.06 ± 0.17 d), cucumber (6.00 ± 0 d), and soybean (5.73 ± 0.30 d) with no significant difference between these three plants, followed by those on pepper and Chinese cabbage (*F* = 7.818, *p* < 0.001) (Figure 1). The developmental duration of first nymphs was the highest on eggplant (2.47 ± 0.14 d), cucumber (2.47 ± 0.14 d), and soybean (2.33 ± 0.21 d) with no significant difference between these three plants (*F* = 4.259, *p* = 0.003). The developmental duration of t second nymphs was the highest on eggplant (2.80 ± 0.15 d), soybean (2.53 ± 0.16 d), and Chinese cabbage (2.37 ± 0.17 d) (*F* = 4.002, *p* = 0.004). The third instar nymphs had the highest developmental duration on soybean (1.10 ± 0.09 d) with no significant difference between five plants (*F* = 0.246, *p* = 0.912). The pupae had the highest developmental duration on cucumber (2.75 ± 0.20 d) with no significant difference between five plants (*F* = 0.114, *p* = 0.977). The developmental duration of adults was highest on soybean (11.43 ± 0.85 d), followed by those on cucumber (9.89 ± 1.26 d), pepper (9.30 ± 1.27 d), and eggplant (8.63 ± 1.37 d) (*F* = 3.497, *p* = 0.011).

The life table of experimental population was established based on the actual observation data of survival rate, female ratio, and amount of eggs laid per female (Table 1). The rates of survival of developmental stages were recorded except for the eggs, because the eggs of *T. flavus* developed in the plant tissues where direct observation was not feasible. The survival rate of second instar nymphs was the highest on soybean and reached 93.33%, followed by those on eggplant, cucumber, Chinese cabbage, and pepper. The third instar nymphs all had a survival rate of >90%, with the exception of Chinese cabbage. The survival rate of pupae was the highest on soybean and reached 96.30%, followed by that on cucumber. In addition, the survival rates of pupae were all >90%, and even reached 100% on Chinese cabbage, eggplant, and pepper. The population trend index (*I*) was >1 and the highest on cucumber followed by those on soybean and pepper. However, females oviposited few eggs on eggplant and Chinese cabbage. The population trend index (*I*) was <1, and the population trend was decreasing on these two plants. Thus, the population exhibited a sustainable growth trend on these three hosts (cucumber, soybean, and pepper) that were more suitable for the development and reproduction of *T. flavus*, while eggplant and Chinese cabbage were not suitable for the oviposition of *T. flavus*.

### 3.2. Population Parameters

The population parameters are listed in Table 2. The intrinsic rate of increase (*r*) was the highest on cucumber (0.1039), followed by those on soybean (0.0966) and on pepper (0.0137). From high to low, the order of generation time was on cucumber (21.9890 d), soybean (20.4760 d), and pepper (19.6270 d). The finite rate of increase (*λ*) on the three plants was all >1, which showed that the *T. flavus* population on all three hosts positively increased. The net reproductive rate (*R*_0_) was highest on cucumber (9.8276 per female), followed by those on soybean (7.2333 per female) and pepper (1.3077 per female). These results indicate that *T. flavus* had higher fecundity or faster development rates on these three plants. In particular, cucumber was the most conducive to reproduction and population growth for *T. flavus*.

### 3.3. Age Stage-Specific Survival Rate (l_x_)

**Figure 2:**
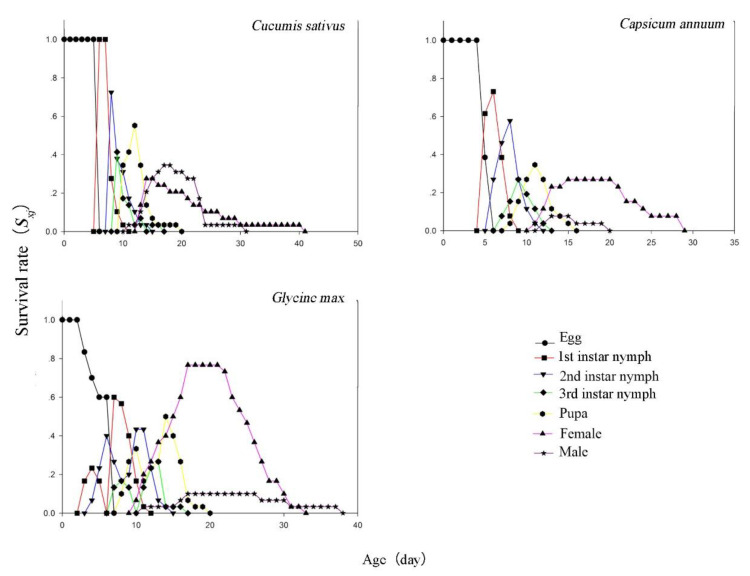
Age stage-specific survival rate (*s_xj_*) of *Thrips flavus* on different plants.

The age stage-specific survival rates of the first nymphs, second nymphs, third instar nymphs, and pupae on cucumber were higher than those on soybean and pepper (Figure 2). However, the survival rate of female adults on soybean (76.67%) was higher than those on cucumber (27.59%) and pepper (26.92%), while the survival duration on soybean (23 d) was shorter than that on cucumber (28 d). For male adults, the survival rate on cucumber (34.48%) was higher than those on soybean (6.67%) and pepper (7.69%). The survival rate of females was longer than that of males on soybean and pepper but not on cucumber.

### 3.4. Age-Specific Survivability and Age Stage-Specific Fecundity

**Figure 3:**
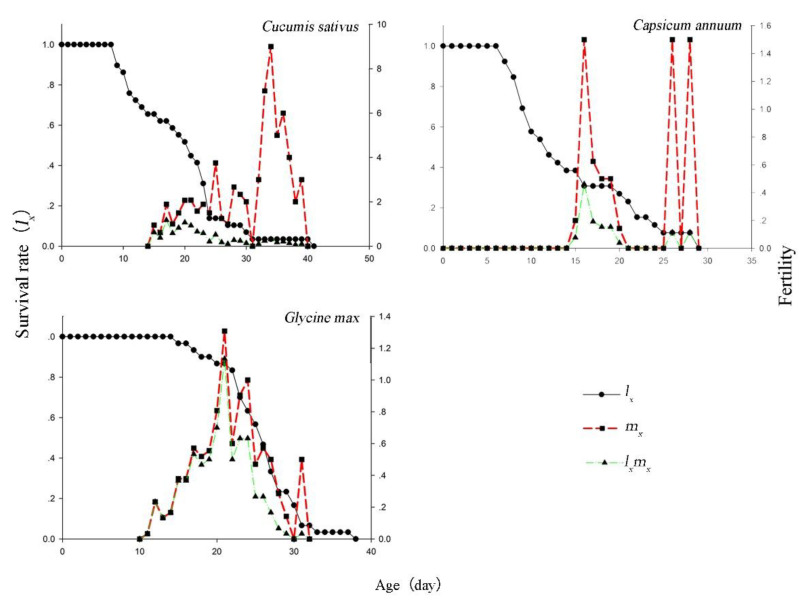
Age-specific survival rate (*l_x_*), age-specific fecundity (*m_x_*), and age-specific maternity (*l_x_m_x_*) of *Thrips flavus* on different plants.

The survival rate was high on three different plants species (Figure 3). The values of age-specific survival rate (*lx*) decreased with age owing to differences in the plant species. The value of *l_x_* indicates that a newly oviposited egg will survive to age (*x*). It was 29.0 d on pepper, 38.0 d on soybean, and 41.0 d on cucumber, respectively. Although the age-specific fecundity (*m_x_*) reached its peak three times within 30.0 d on pepper, *m_x_*, and the age-specific maternity (*l_x_m_x_*) fluctuated a substantial amount. In contrast, the range of change of *m_x_* and *l_x_m_x_* was relatively small on soybean. The peak values of *l_x_m_x_* were 1.13 at 20 d on soybean, 0.46 at 16 d on pepper, and 1.17 at 17 d on cucumber, respectively. This indicated that cucumber could be a suitable plant for oviposition, and soybean could be suitable for the survival of *T. flavus*.

### 3.5. Age Stage-Specific Life Expectancy

**Figure 4:**
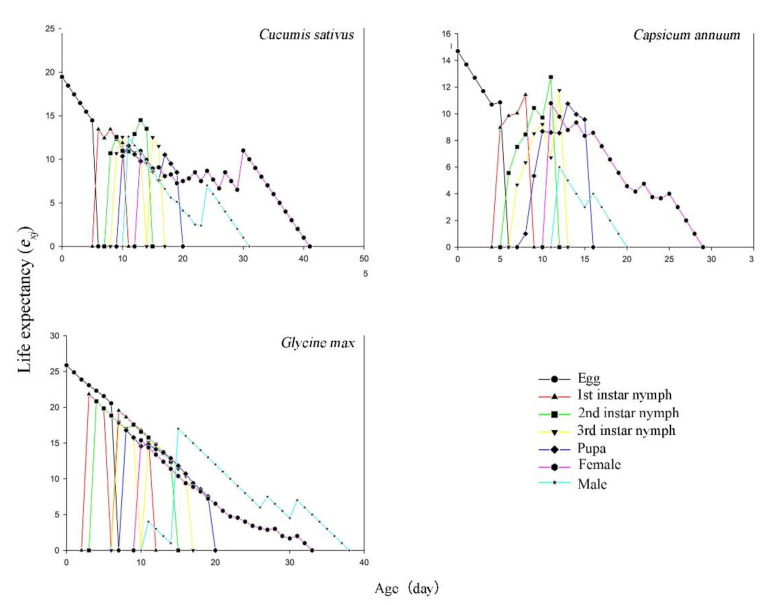
Life expectancy (*e_xj_*) of each age-stage group of *Thrips flavus* on different plants.

The life expectancy (*e_xj_*) is the probability that an individual of age (*x*) and stage (*j*) will survive to age and stage. The life expectancy (*e_xj_*) value of the egg stage decreased. The *e_xj_* values of the other stages first increased and then decreased with age (Figure 4). As shown, female adults on soybean had the highest life expectancy (15.39 d), and the lowest was on pepper (10.79 d). Similarly, the peak value of *e_xj_* for male adults was observed on soybean (17.0 d), and the minimum value was on pepper (5.0 d). This indicated that *T. thrips* has a longer life expectancy on soybean.

### 3.6. Age Stage-Specific Reproductive Value

**Figure 5:**
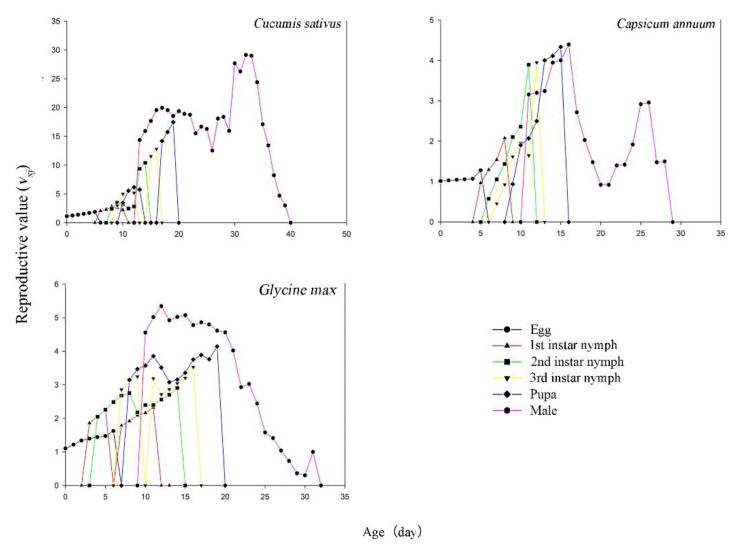
Reproductive value (*v_xj_*) of each age-stage group of *Thrips flavus* on different plants.

The age-stage reproductive value (*v_xj_*) is the contribution of an individual of age (*x*) and stage (*j*) to the future population. The reproductive values (*v_xj_*) first increased and then decreased with the age (*x*) of *T. flavus* (Figure 5). Additionally, the peak *v_xj_* values of a female adult were observed at 16.0 d (4.40/d) on pepper, 17.0 d (19.96/d) on cucumber, and 11.0 d (5.35/d) on soybean.

## 4. Discussion

A life table is an important method to study the mechanism of insect population dynamics [28,29]. The net reproductive rate, intrinsic rate of increase, developmental rate, survival rate, and fecundity can be used to evaluate the adaptability of insects on host plants. The suitability of host plants depends on shorter development times and greater total reproduction [18]. The results showed that five plants had different effects on the different life stages of *T. flavus*. The developmental duration of *T. flavus* was the longest on soybean, followed by that on cucumber. The survival rate of *T. flavus* was the highest on soybean, followed by that on cucumber. The population trend index (*I*) was all >1 and the highest on cucumber, followed by that on soybean (Table 1). However, the intrinsic rate of increase (*r*) and net reproductive rate (*R_0_*) was the highest on cucumber, followed by that on soybean (Table 2). Thus, this indicated that cucumber was the most suitable host for *T. flavus*, followed by soybean and the other three plants. Although *T. flavus* has a wide range of host plants [3,5,6], its selectivity and preference for different host plants varied. In addition, the difference in reproduction on various plants can be used to evaluate their adaptability to specific hosts. In this study, cucumber was suitable for the oviposition of *T. flavus*, and soybean was suitable for their survival. However, eggplant and Chinese cabbage were not suitable for oviposition or survival. This was also found to be the case in *Frankliniella occidentalis* [30].

The relationship between insects and their hosts is highly complex. The strong irritating volatiles or secondary metabolites from Solanaceae plants may affect the selection of *T. flavus* for oviposition. This is similar to the use of some Solanaceae and Cruciferae plants to repel or deter pests, such as *Callosobruchus maculatus*, *Musca domestica*, and *Tetranychus urticae* among others [31,32,33]. With the exception of volatiles or secondary metabolites, the food quality greatly influences the performance of thrips [33]. Plant nutrients can affect the feeding and reproduction of insects, and the adaptability of insects to their hosts, such as *Aeolothrips intermedius*, *Thrips palmi*, and *Thrips obscuratus* [33,34,35,36,37]. In fact, studies have shown that pollen has a substantial influence on the survival rate and oviposition of *F. occidentalis* [38,39,40]. In this study, the leaves of five plants were tested, and the flowering factors of hosts were not considered. *T. flavus* may depend on soybean leaves to maintain its population development in the non-flowering stage. In a previous field study [14,15], we also found that the number of *T. flavus* in the flowering stage was significantly higher than that in the non-flowering stage, which may be a temporary outbreak caused by the flowering stage. *T. flavus* are often found in the flowers of *Ulex* (Fabaceae) in Britain [41]. This indicates that flowers could be an important factor that influences the development of *T. flavus*. The reason could be owing to the nutrients in flowers or their complex structure in which the thrips can easily hide, and this merits further study. Therefore, it is essential to compare the differences in the components of flowers, leaves, and fruits of different hosts, as well as the differences in components of flowers, leaves, and fruits of the same host plant, particularly the factors of flowering and non-flowering [40]. In addition, the leaf structure, leaf thickness, and number of leaf hairs of the host plants may also affect the feeding and oviposition of thrips [42,43]. Future studies may be required to study the feeding behavior of *T. flavus* on different host plants, the way in which host plants are resistant to *T. flavus,* the secondary metabolites in unsuitable plants, and the differences in physiological indices of *T. flavus* after feeding on different hosts.

## 5. Conclusions

This study provides fundamental information on the development duration, experimental population parameters and population growth of *T. flavus* on five plants. *T. flavus* can survive and reproduce on cucumber and soybean, which were two suitable host plants. These results will improve our understanding of population dynamics of *T. flavus* and facilitate the development of more scientific and efficient measures for pest thrips.

## Figures and Tables

**Table 1:** Life table parameters of *Thrips flavus* fed on different plants.

Developmental Stages	Life Table Parameters				
	*Glycine max*	*Cucumis sativus*	*Capsicum annuum*	*Solanum melongena*	*Brassica rapa* var. *glabra*
Egg (%)	–	–	–	–	–
1st instar nymph (%)	100.00	100.00	100.00	100.00	100.00
2nd instar nymph (%)	93.33	76.47	56.25	85.29	73.08
3rd instar nymph (%)	96.43	96.15	94.44	96.55	84.21
Pupa (%)	96.30	76.00	64.71	53.57	62.50
Adult (%)	96.15	94.74	100.00	100.00	100.00
Female ratio	0.87	0.47	0.91	0.38	0.82
Amount of eggs laid per female	8.35	31.67	3.78	0.17	0.92
Amount of egg expected in the following generation (*E*)	603.07	788.01	118.25	2.85	29.02
Population trend index (*I*)	6.03	7.88	1.18	0.03	0.29

**Table 2:** Population parameters of *Thrips flavus* fed on different plants.

Population Parameters	*Glycine max*	*Cucumis sativus*	*Capsicum annuum*
Intrinsic rate of increase (*r*)	0.0966 ± 0.009 a	0.1039 ± 0.024 a	0.0137 ± 0.030 b
Finite rate of increase (*λ*)	1.1015 ± 0.010 a	1.1095 ± 0.026 a	1.0138 ± 0.030 b
Net reproductive rate (*R*_0_)	7.2333 ± 1.139 a	9.8276 ± 4.672 a	1.3077 ± 0.681 b
Generation time (*T*)	20.4760 ± 0.509 a	21.9890 ± 1.400 a	19.6270 ± 3.741 a
Gross reproductive rate (GRR)	10.0600 ± 1.492 b	67.0600 ± 27.688 a	6.4700 ± 2.183 b

Data are presented as mean ± SE. Different small letters in the same row indicate significant differences at *p* < 0.05.

## Data Availability

The data presented in this study are available on request from the corresponding authors.
